# Thyroid Hormone Resistance With a Novel Mutation

**DOI:** 10.7759/cureus.72898

**Published:** 2024-11-02

**Authors:** Kulsum Khan, Alak Moriwala, Zainab Siddiqui

**Affiliations:** 1 Internal Medicine, Nottingham University Hospitals NHS Trust, Nottingham, GBR; 2 Medicine, S. L. Raheja Hospital, Mumbai, IND; 3 Radiology, Dubai Health Authority, Dubai, ARE

**Keywords:** hyperthyroidism, thyroid disorder, thyroid function test, thyroid hormone receptor mutation, thyroid nodule size, thyroid-stimulating hormone (tsh)

## Abstract

Syndrome of thyroid hormone resistance (THR) is a rare inherited condition characterized by a reduced responsiveness of the tissues to the thyroid hormone. The syndrome is caused primarily by mutations in the thyroid hormone receptor beta (THRB) gene, leading to impaired hormone receptor function. It is a diagnosis of exclusion and often leads to delays in establishing the diagnosis. Management is usually conservative, as over-treating can be unnecessary and potentially detrimental. Our case report aims to highlight the changes in thyroid function tests and the subtle presenting symptoms of this disease so that clinicians are more mindful of this rare condition. It brings to attention the importance of follow-up to monitor the lab values and reach an accurate diagnosis. We also report a novel mutation identified in the THRB gene.

## Introduction

Syndrome of thyroid hormone resistance (THR) is a rare inherited condition characterized by a reduced responsiveness of the tissues to the thyroid hormone. The causes of hormone resistance can be vast, potentially affecting the thyroid hormone receptor or other unidentified steps in the hormone action pathway [1]. It is generally caused by germline mutations of the thyroid hormone receptor beta (THRB) gene [2]. It presents with high serum concentrations of T3 and/or T4, along with a normal or slightly elevated thyroid-stimulating hormone (TSH) concentration. Symptoms are often non-specific, which makes it challenging for this condition to be diagnosed and can potentially lead to a delay in diagnosis. The goal of treatment is to maintain a normal serum TSH level and a euthyroid state in addition to offering genetic counseling [3].

This article was previously presented as a poster at the 11th EDEC on 4th March 2021.

## Case presentation

**Figure 1:**
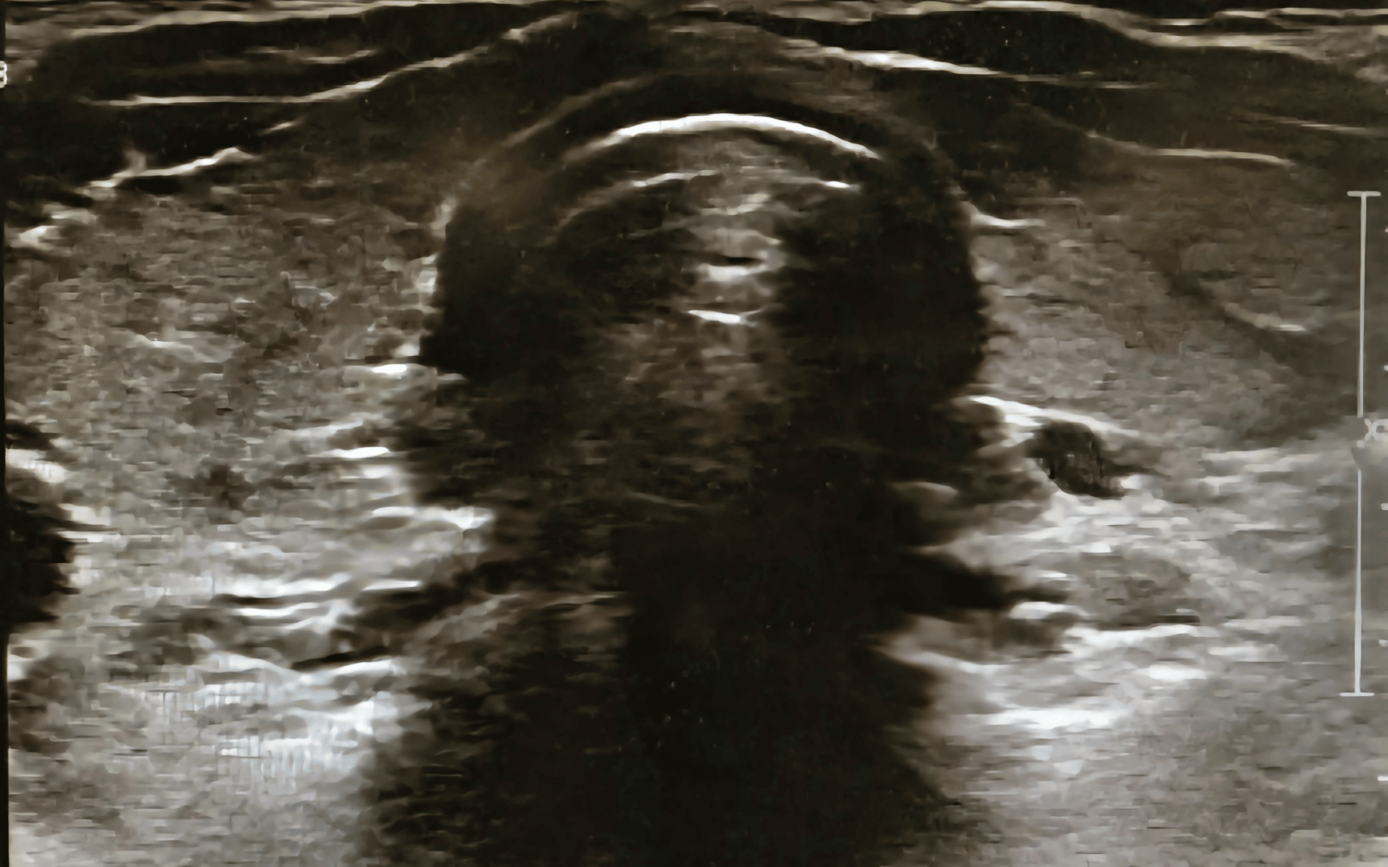
Diffuse heterogeneous echotexture of the thyroid gland and a posterior left lobe nodule.

A 28-year-old previously healthy female presented to our endocrinology outpatient department after she was diagnosed with mixed hypo/hyperthyroidism in another facility based on deranged thyroid function tests. She was prescribed levothyroxine, which she was not adherent to. She complained of intermittent cold intolerance and hand pain but was otherwise asymptomatic. Physical examination was unremarkable. Lab tests revealed an elevated TSH and free T4 along with positive thyroglobulin and anti-thyroid peroxidase antibodies. A thyroid ultrasound was done and revealed an average-sized thyroid gland with diffuse heterogeneous echotexture suggestive of thyroiditis along with a posterior left lobe nodule (Figure 1).

An initial diagnosis of Hashimoto’s thyroiditis was made based on the above, with advice to follow up in three months and a repeat of thyroid function tests.

Subsequently, follow-up labs yet again revealed elevated TSH and T4 thus raising the suspicion of thyroid hormone resistance. A thyroid hormone resistance panel was sent. The result of the THRB mutation analysis was positive for a heterozygous variant of uncertain significance (VUS) in the THRB gene. It also identified a mutation affecting the first base in exon 5 of the THRB gene.

Since she was asymptomatic, she was not started on treatment and was advised for regular follow-up. Currently, she is on regular follow-up with thyroid function tests and remains asymptomatic.

## Discussion

Thyroid hormone resistance has been detected in one of 40,000 live births and a variety of mutations have been identified. It is characterized by a reduced responsiveness of tissues that express the thyroid receptor to the actions of T3 due to mutations in the thyroid receptor gene. Although two genes of the thyroid receptors exist, i.e., alpha and beta, defects are more commonly observed in the beta receptor genes. As a result, this leads to elevated, non-suppressed levels of TSH with elevated levels of T3 and T4 [2].

A feature of resistance to thyroid hormone (RTH)-beta is the relative sparsity of symptoms and signs of thyroid dysfunction despite the presence of high serum T4 and T3 concentrations. Most patients with THR are euthyroid and clinically symptom-free. However, goiter, hyperactivity, and tachycardia are the clinical findings that often lead to the evaluation of thyroid function. Rarely, patients may also present with symptoms of hypothyroidism, such as in our case where she complained of some cold intolerance [4].

The subsequent findings of high T4 and T3 often result in an inappropriate diagnosis of hyperthyroidism and unnecessary treatment with antithyroid medications. An important differential to consider is TSH-secreting pituitary adenoma, which presents biochemically with a similar profile. It may be distinguished by a pituitary MRI (abnormal in TSH adenoma) and measurement of glycoprotein alpha subunit to TSH ratio (elevated in TSH adenoma). Similarly, other differentials include increased serum-binding proteins, familial dysalbuminemic hyperthyroxinemia, the presence of anti-iodothyronine (anti-T4, anti-T3) antibodies, or certain drugs such as amiodarone and beta-blockers [5].

Furthermore, our patient’s next-generation sequencing (NGS) analysis identified a heterozygous missense and putative splice variant c.284G>Cp. (Gly95Ala) affecting the first base of exon 5, which, to the best of our knowledge, has not been described in the literature so far. According to the literature, most cases of THR-beta are caused by mutations in exons 8, 9, and 10 [2].

## Conclusions

We have described a case of a young female with THR syndrome and have identified a novel mutation in the THR-beta gene, which is an alteration in the genetic sequence of the gene. It is a complex and rare endocrine disorder requiring a nuanced approach to diagnosis and management. It is vital to keep THR as one of the differentials in patients presenting with a combination of elevated TSH and T4 levels as failure to recognize may lead to incorrect treatment with anti-thyroid medications. Management of THR should be based on patients’ clinical picture rather than their biochemical abnormalities. Fortunately, most patients are symptom-free and do not require treatment.

Understanding the genetic basis and variable clinical presentation is essential for providing tailored care to the affected individuals. Ongoing research continues to unravel the molecular mechanisms of the syndrome and identify potential therapeutic targets for the condition.
